# The health and economic benefits of reducing intimate partner violence: an Australian example

**DOI:** 10.1186/s12889-015-1931-y

**Published:** 2015-07-09

**Authors:** Dominique A. Cadilhac, Lauren Sheppard, Toby B. Cumming, Tharshanah Thayabaranathan, Dora C. Pearce, Rob Carter, Anne Magnus

**Affiliations:** Department of Medicine, Translational Public Health Unit, Stroke & Ageing Research, School of Clinical Sciences, Monash University, Monash Health Research Precinct (MHRP) Building, Level 1, 43-51 Kanooka Grove, Clayton, 3168 VIC Australia; Stroke Division, Florey Institute of Neuroscience and Mental Health, 245 Burgundy St, Heidelberg, 3084 VIC Australia; Deakin Health Economics, Deakin University, Burwood, 3125 VIC Australia; Melbourne School of Population and Global Health, Faculty of Medicine, Dentistry & Health Sciences, The University of Melbourne, 3010 Melbourne, Australia

**Keywords:** Australia, Female, Intimate partner violence, Cost-benefit analysis, Health care costs, Economic models, Mortality, Quality-adjusted life years, Risk reduction behaviour

## Abstract

**Background:**

Intimate partner violence (IPV) has important impacts on the health of women in society. Our aim was to estimate the health and economic benefits of reducing the prevalence of IPV in the 2008 Australian female adult population.

**Methods:**

Simulation models were developed to show the effect of a 5 percentage point absolute feasible reduction target in the prevalence of IPV from current Australian levels (27 %). IPV is not measured in national surveys. Levels of psychological distress were used as a proxy for exposure to IPV since psychological conditions represent three-quarters of the disease burden from IPV. Lifetime cohort health benefits for females were estimated as fewer incident cases of violence-related disease and injury; deaths; and Disability Adjusted Life Years (DALYs). Opportunity cost savings were estimated for the health sector, paid and unpaid production and leisure from reduced incidence of IPV-related disease and deaths. Workforce production gains were estimated by comparing surveyed participation and absenteeism rates of females with moderate psychological distress (lifetime IPV exposure) against high or very high distress (current IPV exposure), and valued using the friction cost approach (FCA). The impact of improved health status on unpaid household production and leisure time were modelled from time use survey data. Potential costs associated with interventions to reduce IPV were not considered. Multivariable uncertainty analyses and univariable sensitivity analyses were undertaken.

**Results:**

A 5 percentage point absolute reduction in the lifetime prevalence of IPV in the 2008 Australian female population was estimated to produce 6000 fewer incident cases of disease/injury, 74 fewer deaths, 5000 fewer DALYs lost and provide gains of 926,000 working days, 371,000 days of home-based production and 428,000 leisure days. Overall, AUD371 million in opportunity cost savings could be achievable. The greatest economic savings would be home-based production (AUD147 million), followed by leisure time (AUD98 million), workforce production (AUD94 million) and reduced health sector costs (AUD38 million).

**Conclusions:**

This study contributes new knowledge about the economic impact of IPV in females. The findings provide evidence of large potential opportunity cost savings from reducing the prevalence of IPV and reinforce the need to reduce IPV in Australia, and elsewhere.

**Electronic supplementary material:**

The online version of this article (doi:10.1186/s12889-015-1931-y) contains supplementary material, which is available to authorized users.

## Background

Intimate partner violence (IPV) is an important global public health problem. It was ranked 23^rd^ in terms of Disability Adjusted Life Years (DALYs) arising in women in the most recent update of the Global Burden of Disease (GBD) Study 2010, following after other important risk factors such as high total cholesterol, suboptimal breastfeeding, alcohol use, physical inactivity, high blood pressure and dietary risks [1].

Globally, IPV is a major public health concern because it is the most common form of violence that women experience [2]. IPV has been defined for an international survey as women who had a current or former intimate partner and had experienced violence from that partner during their lifetime [3]. Alternate terminologies include *domestic violence, family violence or relationship violence.* IPV refers to violence occurring between people who are, or were formerly, in an intimate relationship and were subject to economic, psychological or emotional abuse through to physical and sexual violence [4].

There have been several attempts to make meaningful cross-country comparisons in rates of IPV [2, 5–10]. One of the most comprehensive (including past and current exposure) to compare rates of IPV across countries was the *International Violence Against Women Survey* (IVAWS). One of the main aims of the IVAWS was to facilitate international comparisons by using standardised measurement tools and techniques to establish prevalence in each participating country. Johnson et al. [3] reported data for Australia and eight other countries. Prevalence of IPV was highest in Mozambique (40 %) and lowest in Hong Kong (9 %), with other countries in between: Czech Republic (37 %), Costa Rica (36 %), Australia (27 %), Denmark (22 %), Poland (16 %), Philippines (10 %) and Switzerland (10 %) [3]. In 2010, Devries and colleagues estimated women’s lifetime prevalence of IPV for 21 regions [2]. Globally, 30 % of women aged 15 and over were reported to have experienced IPV during their lifetime. In this latter study,  the greatest prevalence of IPV was found in Central Sub-Saharan Africa (66 %) and lowest in East Asia (16 %), while, Australasia had an IPV prevalence of 28 % [2]. In the most recent personal safety survey for Australia, 17 % of women surveyed in 2012 had experienced IPV since the age of 15 [11]. In Victoria (Australia), IPV contributes 3 % to the total disease burden and 8 % in women aged 15–44 [4]. In this younger age group, it is the leading contributor to death and disability, being responsible for more disease burden than other well-known risk factors, such as high blood pressure, smoking and obesity [4].

Recognizing its importance on health and production consequences, the prevention of IPV should be a priority in all countries [12]. A reduction in prevalence of IPV can have important impacts on society, including enhanced psychological well-being, which at a population level improves general health and productivity. Quantifying the economic impact of IPV is also important, since it demonstrates the extent of these societal impacts. However, few studies of the costs of IPV have been undertaken, and the methods used vary since the research questions also differ e.g. including impacts of IPV on both men and women, including a larger range of indirect or intangible costs such as counting the costs to future generations, costs of pain and suffering, consumption related costs, or transfer costs [5]. This means that these studies are generally incomparable due to the different definitions and assumptions applied (including the time horizon for counting the costs), limiting the ability to compare the economic implications of IPV with other important conditions. Previous costing studies in Australia have found IPV was responsible for increased costs to the community between AUD8.1 billion and AUD9.9 billion (2007–08 dollars) annually [5, 13] and was predicted to grow to AUD15.6 billion by 2021–22 if no action is taken [13]. These earlier studies provide estimates of the total economic costs that could be avoided if the violence was to be eliminated, rather than estimating the extent of societal benefits if the prevalence of IPV against females was to be reduced by a **feasible** amount.

The aim of this study was to separately quantify the health and economic benefits within the paid and unpaid production sectors and health sector that could be achieved following a feasible reduction (as opposed to a complete elimination) of IPV in the Australian female adult population. This study on IPV was part of a larger study funded by the Victorian Health Promotion Foundation (VicHealth) whereby the benefits of feasible reductions in the prevalence of long term harmful alcohol consumption, physical inactivity, high body mass index, tobacco smoking, inadequate consumption of fruit and vegetables, and IPV were estimated using a standardized approach [14]. Therefore, the advantage of our study in relation to prior research in this field is that we can reliably put the findings for IPV within a broader policy context. This is because we  highlight not just the total attributable burden based on the aspects we have measured, but also what could be gained if a realistic absolute reduction in IPV prevalence could be achieved.

## Methods

Relevant diseases and injuries associated with IPV include depression, anxiety, cancer, chronic obstructive pulmonary disease (COPD), and suicide [15, 16]. To estimate the health and economic benefits to society of reducing the prevalence of IPV, the impact of an absolute reduction in the prevalence of IPV levels for the 2008 Australian female population was calculated. This was quantified as reduced: a) incident cases of preventable IPV-related diseases/injuries; b) deaths; and c) disability adjusted life years (DALYs). We simulated benefits of risk factor prevalence reductions for this one reference year. However, the time horizon for benefits estimation was based on a lifetime perspective for that 2008 female population cohort.

The selected feasible prevalence reduction targets for IPV in Australia were based on consensus by an expert Advisory Committee who were presented with a review of the literature. Since the literature on prevention interventions did not include readily usable estimates for feasible reductions, prevalence rates of IPV from other countries were consulted.

Comparing prevalence rates in Australia to other countries provides indirect evidence of possible achievable prevalence levels from a country with a broadly comparable social, demographic and economic profile [14]. The feasible reductions could then be modelled against attainment of lower prevalence levels of IPV as observed in a comparable country to Australia (referred to as an ‘Arcadian’ normal [17]). However, comparing across countries posed some difficulties since some prevalence estimates were based on all women, others based on only those women who have ever had a partner, and others based on women who currently have a partner. Furthermore, the definition of an ‘intimate partner’ (husband, de facto, cohabiting partner, boyfriend, etc.) and the definition of what constitutes violence differs across studies. Using data from the IVAWS study overcame many of these issues [3]. However, we acknowledge that in this study there were important differences between participating countries, including sample size, age distribution of respondents, response rate and method of interview. Among the countries with lower prevalence estimates than Australia, Poland, Philippines and Hong Kong were ruled out as comparators for being socio-culturally different. Although broadly comparable, Switzerland had a prevalence of only 10 % which seemed remarkably low, and was considered by the Advisory Committee too ambitious a target for Australia to achieve in the short term. Denmark, with a prevalence of 22 %, was selected as the most appropriate Arcadian comparator. The Advisory Committee agreed that there was the potential to reduce IPV prevalence in Australia by up to 5 percentage points, shifting the estimated current prevalence of 27 % to 22 %. A 5 percentage point absolute reduction in IPV was selected as an ideal feasible target (22 %), with a progressive target of 24.5 % also assessed.

Past and current exposure to IPV is most influential in determining the current health burden, health sector costs and production and leisure costs, but past exposure is not amenable to prevention efforts. The effects of past exposure can only be ameliorated through current health services. It is current levels of IPV that are amenable to preventive interventions and, thus gradually over time, the lifetime prevalence of IPV can be expected to fall. If a woman ceases to be exposed to current IPV, we have modelled that she will change from a high level of psychological distress to a moderate level, since she remains exposed to lifetime (past) IPV.

### Data sources for simulation models

The most relevant Australian data sources for our reference year 2008 were used. The current estimates for the lifetime prevalence of IPV by age for females were based on the 2003 Burden of Disease incidence and deaths data because these were the most current data available at the time this study was undertaken. Population Attributable Risk Fractions (PAFs), and DALYs were also obtained using the 2003 Australian Burden of Disease data files [16] made available for this study. The definition for IPV in the 2003 Burden of Disease study was based on two categories of exposure to IPV, which included physical or sexual violence by a partner in the last 12 months, and physical or sexual violence by a partner more than 12 months ago. This was estimated, using information from the Women’s Safety Survey data reported by the Australian Bureau of Statistics (ABS), to determine the prevalence of ‘IPV without child sexual abuse’ and ‘child sexual abuse and IPV combined’ where an adjusted relative risk to account for the combined exposure state of having experienced both child sexual abuse and intimate partner violence was used [16]. Begg et al. only provided estimates for females due to insufficient evidence on prevalence and risk among males.

Since the Australian National Health Survey (NHS) 2004–05 does not measure IPV directly, we used levels of psychological distress as a proxy for exposure to IPV. This is because psychological conditions including depression and anxiety, as well as suicide and self-inflicted harm contribute most (74 %) of the disease and injury burden associated with IPV [18]. The population groups compared were the female Australian population reporting high or very high levels of psychological distress (score 22–50 on the Kessler Psychological Distress Scale −10), and the females reporting moderate levels of distress (score 10–21) [19, 20]. We used the proxy distress levels to isolate workforce behaviours (workforce participation, absenteeism and days out of role), since females with diagnosed depression and anxiety were not identified in the NHS. Demographic data, employment status, and health-related actions of females in the comparator groups were also obtained from the 2004–05 NHS dataset (Table 1).

**Table 1:** Demographics and days of high psychological distress due to ill health by age, sex and work force status for the 2008 female adult Australian population

Females	High or very high distress	Moderate distress
Age summary		
Age 15–64 y	989,848	1,636,652
N (95 % CI)	(927,959–1,051,738)	(1,556,018–1,717,285)
Age 65+ y	153,190	300,836
N (95 % CI)	(132,009–174,372)	(265,299–336,372)
Age 15+ y	43.8	43.5
Mean (95 % CI)	(42.7– 44.9)	(42.8– 44.2)
In Labour Force (15+ years)^a^		
% (95 % CI)	53 % (50–57 %)	62 % (59–64 %)
Mean days off work (95 % CI)	0.69 (0.52–0.85)	0.28 (0.21–0.35)
Not in Labour Force		
% (95 % CI)	47 % (43–50 %)	39 % (36–41 %)
Mean days of reduced activity: 15–64 y (95 % CI)	3.03 (2.54–3.53)	1.47 (1.15–1.79)
Aged 65+ years		
% (95 % CI)	13.4 % (11.7–15.3 %)	15.5 % (13.9–17.3 %)
Mean days of reduced activity (95 % CI)	3.62 (2.70–4.55)	2.25 (1.75–2.75)

Source: National Health Survey 2004–05 (ABS, 2006). Mean days measured over a two week period

*CI* confidence interval, *N* number

^a^includes unemployed seeking work and 65+ years

The 2000–01 Disease Costs and Impact Study Excel files, which adopted the Burden of Disease classification system, were used to estimate the change in health sector costs from reduced incidence of disease and injuries associated with IPV [21, 22]. Household production and leisure time were derived from the 2006 ABS Time Use Survey [23]. Current average wages for 2008 were sourced from the ABS and published government pay scale summaries [24, 25].

### Data analyses

Details of the simulation models used in the current study have been published in detail elsewhere [14, 26–28]. Briefly, the net differences in mortality, incident morbidity and consequent health sector costs and the impacts on paid and unpaid production and leisure between the current prevalence of IPV and the two target prevalence levels for the 2008 Australian female adult population were estimated with population-based simulation models developed in Excel (Microsoft Corporation, 2003). Cost data from other years were adjusted to 2008 Australian dollars (AUD) by applying health price inflators [29]. For readers wishing to convert AUD in 2008 to US dollars in 2008, the purchasing power parity of AUD1.48 should be used [30]. A 3 % discount rate for lifetime benefits was applied [31], and varied in sensitivity analyses using 0, 5 and 7 % (data not reported but available from the authors).

To estimate changes in ***health sector costs***, the attributable portion of total health sector costs to disease and injuries associated with IPV were estimated using PAFs for diseases and injuries attributable to IPV for females by age group [16]. The modelling of lifetime health expenditure cost savings from these data was estimated by assuming the annual health sector costs of treating incident and prevalent cases of disease attributable to IPV approximates the life time health sector cost savings of a reduction in the incident cases of IPV for our reference (2008) population.

If a target reduction in IPV prevalence was achieved, the ***production gains/losses*** and ***taxation effects*** in the Australian economy were modelled by the simulation of a theoretical cohort of Australian women (ages 15–65 years) during their working years until retirement age [14]. Briefly, the working lifetime income earned and taxation paid by females were calculated taking into account known participation rates and absenteeism rates by age of the exposed (high distress) and the comparator (moderate distress) populations and average female wage rates. The production gains or losses arise from changes in income earned and taxation paid that result from fewer deaths and incident cases of disease and injury, associated with the reduction in prevalence of IPV in the female adult population. In a separate sensitivity analysis, unisex average wage rates were applied to the total female population (analysis available from the authors).

Two methodological techniques were used to value the production gains or losses. First, the Friction Cost Approach (FCA) was used where it was assumed that individuals who die or leave the workforce due to disability would be replaced after a specified friction period; we used 3 months and varied this to 6 months in a sensitivity analysis. The second valuation technique of Human Capital Approach (HCA) was used to value workforce production gains or losses. The HCA counts as lost all the future income up to age 65 from an individual who leaves the workforce due to death or disability. There remains debate in the economic literature about which method is preferable [32, 33] since they give such divergent results and measure different constructs. The HCA measures the potential productive value of a human life, whereas the FCA measures the shorter term losses faced by business and individuals when an unplanned departure from the workforce occurs. For the purposes of the current study, the FCA provided a stronger logical connection to the actual likely cost impact of premature death or disability associated with IPV on industry and thus only FCA results are presented in this paper [14].

The economic value of hours of lost leisure and household production associated with diseases attributable to IPV was estimated using a separate ‘Household Production and Leisure Time’ model [14]. The NHS 2004–05 provided self-reported days out of role for the exposed (very high to high distress) and comparator (moderate distress) Australian women. The net difference in the value of the days out of household production and leisure time between the comparator groups was counted as the potential economic gain. Impacts on both working and non-working women by age were estimated and household production was defined as the hours spent performing non-paid household duties such as cooking, shopping, cleaning, child care and maintenance. These were valued at ‘replacement cost’ using the average 2008 market based wage rates for domestic services and child care. Leisure time comprised social and community interaction, together with recreation and leisure activities only and was valued using the ‘opportunity cost method’, applying one third of the average female 2008 weekly earnings [24].

### Uncertainty analyses

Multivariable probabilistic uncertainty analyses were undertaken using @RISK software version 4.5 for Excel (Palisade Corporation, Ithaca, New York 2005). Input variables were modelled as known distributions rather than single values where uncertainty existed (e.g. each surveyed parameter and life-years remaining). Uncertainty in wages, participation rates and absenteeism were captured in the reported survey standard errors [19, 25, 34]. Uncertainty in health outcomes was not incorporated directly, because the reported 2003 DALY estimates attributable to IPV included no uncertainty ranges. Instead we relied on varying the absolute IPV prevalence reduction in a sensitivity analysis to convey variability around improved health outcomes. The range of values that were simulated as part of uncertainty and sensitivity analyses are available in an additional file. In this Additional file 1: Table S1 contains the input values (average, low and high) of child care, domestic services and average weekly earnings used to estimate household production and leisure time costs and Table S2 provides a summary of input parameters and uncertainty ranges for the economic models and contains detail of the sources, values, uncertainty distributions and detailed comments relevant to all data inputs used in the modelling see Additional file 1: Table S1 and S2. For example, we varied work force participation rates, absenteeism rates, average weekly earnings, days worked in a year, days of reduced activity due to ill health and years of remaining life expectancy as part of these analyses. Monte Carlo sampling with minimum 4000 simulations were used to estimate a mean and 95 % uncertainty interval for the outcome parameters.

## Results

**Fig. 1:**
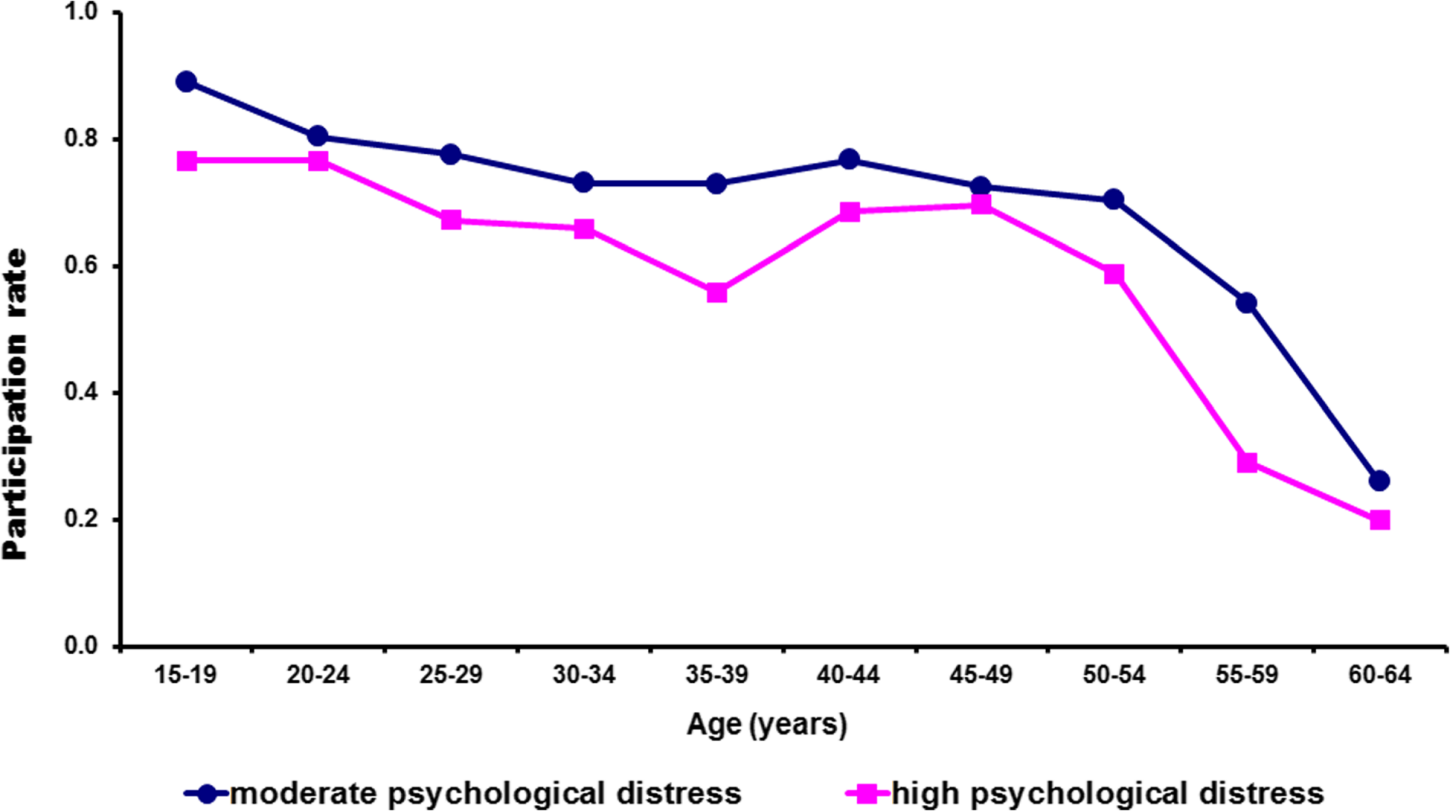
Workforce participation rates of women with high levels of psychological distress compared to women with moderate levels of distress by age. Source: adapted from data obtained from National Health Survey 2004–05 [19]

The demographic data and days of reduced activity for females with moderate psychological distress and high or very high distress by age and workforce status are presented in Table 1. Females with high or very high distress participated less in the workforce than females with moderate distress (Fig. 1). The females with high or very high distress in the workforce, took more days off work compared with females with moderate distress. In addition, females with high or very high distress not in the workforce or past retirement age had more days of reduced activity compared to females with moderate distress.

If the prevalence of IPV in the adult Australian population was reduced by 5 percentage points, the estimated 34,000 annual new cases of IPV-related disease could be reduced by 6000 (18 %); the 440 annual deaths attributed to IPV could be reduced by 74 deaths (17 %); and the estimated 29,000 DALYs attributed to IPV could be reduced by about 5000 (17 %) (Table 2). Nearly half of these benefits would arise if a 2.5 percentage point progressive absolute reduction in prevalence target was achieved.

**Table 2:** Health status and production effects from reducing the prevalence of intimate partner violence

Benefit	Feasible 5 percentage point absolute prevalence reduction target		
		95 % uncertainty interval	
	Mean (‘000 s)	Lower Limit (‘000 s)	Upper Limit (‘000 s)
Disability adjusted life years	5	n/a	n/a
Incidence of intimate partner violence related disease	6	n/a	n/a
Mortality	0.07	n/a	n/a
Lifetime			
Leisure (days)	428	296	568
Absenteeism (days)	926	n/a	n/a
Days out of home based production role (days)	371	259	491
Early retirement (persons)	0.52	n/a	n/a

Incidence of disease and mortality calculated for all age groups. Leisure and home based production calculated for persons aged 15+ years. Absenteeism and early retirement calculated for persons aged 15–64 years. All estimates uncorrected for potential joint effects of multiple risk factors. Note the result for absenteeism has been updated since in the Full Technical Report this figure had not been discounted and was incorrect (http://www.vichealth.vic.gov.au/~/media/ResourceCentre/PublicationsandResources/Knowledge/Research%20Report_FINAL_July09.ashx, last accessed 13 June 2015)

*n/a,* not available, unable to be estimated based on the data that were available

**Fig. 2:**
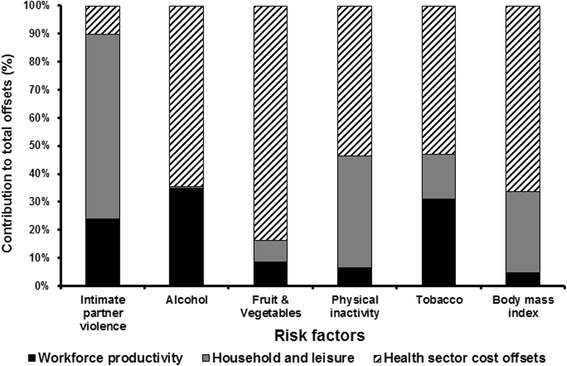
Proportion of opportunity cost savings from reductions in various risk factors by economic category

The estimated health and economic benefits from reduced IPV resulted in total potential opportunity cost savings of AUD377 million, which is comprised of AUD38 million to the health sector (19 % of IPV attributable annual health sector costs), AUD94 million in workforce production (FCA), AUD98 million in leisure based production and AUD147 million in home based production (Table 3). The latter production costs represent approximately 18 % of the total attributable to this risk factor (AUD1,801 million). The largest component of these total potential opportunity cost savings would occur in household production and leisure (Fig. 2).

**Table 3:** Economic outcomes from reducing the prevalence of intimate partner violence

Economic outcomes	Feasible reduction target^a^		
		95 % uncertainty interval	
	Mean (AUD million)	Lower limit (AUD million)	Upper limit (AUD million)
Health sector costs	38	n/a	n/a
Production Costs FCA	88	29	185
Recruitment and training costs	6	n/a	n/a
Taxation effects FCA^b^	16	4	36
Leisure based production	98	61	144
Home based production	147	102	195
Total production FCA^c^	377	227	461
Sensitivity analysis			
Production Costs HCA	434	253	621
Taxation effects HCA^b^	57	29	87
Total production HCA^c^	678	480	884

All estimates uncorrected for joint effects of the presence of multiple risk factors in individuals. Health sector, leisure and home based production based on females 15+ years. Production, recruitment and training and taxation effects based on females 15–64 years

*HCA* human capital approach, *FCA* friction cost approach (preferred conservative estimate), *n/a* not available, unable to be estimated based on the data that were available

^a^These are not estimates of immediately realizable cash savings

^b^Taxation is treated as a transfer payment and should not be added to production effects

^c^Total production is the sum of workforce production costs, household- and leisure-based production

## Discussion

The primary finding of this study is that a feasible 5 percentage point absolute reduction in prevalence of IPV can lead to total potential opportunity cost savings of AUD377 million, with only 10 % of the savings arising in the health sector. The largest potential saving from reduced lifetime prevalence of IPV in the female population will occur in home based production, followed by leisure, and workforce production. These savings would be much larger (approx. AUD2 billion) if IPV was able to be totally eliminated from Australia (AUD207 million in health sector, AUD1,801 million [FCA] in production and leisure). However, our aim was to identify savings that were realistic and relevant to the setting of future public health campaigns focusing on risk factor reduction strategies.

An important aspect of the present study was the separate identification of benefits across workforce, household production and leisure time associated with reduced IPV. Earlier studies that have not accounted for home-based and leisure production underestimate potential savings in the context of IPV prevention. While the current methodology does identify the contribution of household production and leisure, it is conservative since it excludes an assessment of the additional time available for household and leisure tasks, which the cohort of the working age 2008 female population will have following retirement, through their remaining life expectancy.

We are able to compare results of reducing IPV with five other risk factors assessed using the same methodology and data sources [14]. We found that the total economic benefit of reducing IPV was ranked third behind gains following reductions in high risk alcohol consumption and tobacco consumption. This is jointly explained by the different reduction targets deemed feasible for each respective risk factor, the distribution of the population exposure to the risk factors across age groups and sexes as well as the disease and injuries associated with risk factors. While we recognise a potential equity issue by applying lower average wage rates for females compared with males, we were able to test its impact in sensitivity analysis. Adopting annual average unisex wages increased the overall estimates IPV gains by AUD25 million. However if we had analysed and reported all the risk factors with unisex wage rates, the relative ranking of the gains through a feasible reducing IPV would still rank third behind the gains following feasible reductions in alcohol consumption and tobacco use.

The distribution of opportunity cost savings differs for IPV relative to other risk factors we have evaluated using the same methodology and data sources [14]. For all others, the savings in the health sector represent the larger proportion of total savings (Fig. 2). Health sector cost savings due to reduced prevalence of IPV were only found to be 10 % of the total savings. In all other risk factors we evaluated (except physical inactivity), the opportunity cost savings to the health sector were as high as the productivity and leisure costs, if not two to three times greater [14]. This arises primarily due to four main reasons: (i) the benefits of reducing IPV apply mostly to the subset of females who are providing the child bearing and child raising roles during their productive years; (ii) there are disproportionately lower rates of treatment of mental illness associated with IPV compared to physical impacts of cancers and heart disease associated with other major risk factors, (iii) only 41 % of women with any mental health problem (assessed as lifetime prevalence and 12 months symptoms prior to the survey date) sought help for their mental health problem as reported in the most recent National Mental Health Survey [35]; and (iv) a General Practitioner was likely to be the service provider most frequently sought by females with a mental health problem, hence there is an absence of expensive hospital costs in the treatment of anxiety disorders and depression [35].

Comparisons with other IPV research in this field are difficult because of differences in methods, assumptions, populations included and the scope of the costs analyzed. However, earlier assessment of the costs of domestic violence in Australia also highlighted that household, leisure and production impacts are where the greatest costs attributable to IPV may be found [5].

Our estimates are very conservative in comparison to other studies where estimates of potential cost savings to future generations of children in families experiencing domestic violence are included. In the studies reported by Access Economic [5] and KPMG, second generation costs of AUD28 million were to be expected from a 10 % reduction in IPV prevalence [13]. This category includes short-term costs of providing protection and other services (such as child protection services, childcare and remedial/special education) to children of relationships where there is domestic violence, and longer-term costs imposed on society by these children as they grow older (such as increased crime and future use of government services). Our estimates, which take a narrower perspective and address realistic reductions in prevalence, are an underestimate of total current and future generation societal costs. In addition, by excluding males subjected to IPV (in 2012 estimated to be 5.3 % of Australian males experiencing physical or sexual abuse since the age of 15 by a partner) [11], we have understated the potential economic impacts of reducing the prevalence of IPV.

Several other limitations of the current study must be acknowledged. Since women are not directly questioned in the NHS on the issue of IPV, we have relied on surveyed levels of psychological distress amongst surveyed females as a proxy. We acknowledge that not all women with psychological distress, as measured on the Kessler 10, are IPV victims, and nor are women with psychological distress confirmed as having psychological conditions. Since direct evidence was unavailable from the NHS for women with IPV, we chose to use high psychological distress as a reasonable proxy, however this may have led to sources of under- or overestimation, of workforce impacts, since it remains unclear if women who experience IPV have similar patterns of psychological distress as women who do not. The Burden of Disease studies have provided evidence  that depression and anxieties are the largest components of illness attributable to IPV [36]. For this study, the levels of depression and anxieties was adequately captured by the Kessler 10 score used in the NHS [20]. To-date, there remains a lack of consistent and direct evidence for IPV in Australia and the ABS has only recently released a national data framework for enabling a standardized approach to capturing data on family, domestic and sexual violence, so in the future more direct evidence may be available for these studies [37]. Another potential limitation is that we have not controlled for the presence of other risk factors, or socioeconomic status, in the analysis and this may be a source of potential over- or under-estimation of benefits. We have assumed risk reversibility and risk reduction arising from absence of current IPV, and that the cross sectional evidence in the NHS related to workforce behaviours would be associated with a future change in IPV status of women. Longitudinal cohort data are missing at this point to confirm our assumptions. Despite these limitations, we attempted to address such sources of under- or over-estimation by undertaking detailed sensitivity and uncertainty analyses.

## Conclusions

Investment in disease prevention and health promotion in Australia is dwarfed by avoidable spending on disease treatment. The findings of this study contribute important new knowledge about the major impact of IPV on the productivity of females in both the paid and unpaid sectors, as well as health sector expenditure. Our findings reinforce the argument that greater investment in IPV reduction strategies is required and economically justified, particularly in young women. Importantly, our study highlights the need for better data in this field so that the economic impacts can be assessed using direct evidence. We applaud the ABS and the Australian Government who have now established a national data framework to address these methodical issues [38]. While anxiety and depression represent the greatest proportion of the disease burden attributable to IPV, women subjected to IPV tend to have other behavioural risk factors for chronic disease and the impacts extend to the whole family, in particular children who can also suffer health impacts from witnessing IPV. Our findings provide a much needed business case to support policy makers in addressing this important societal issue.

## Additional file

Additional file 1: Table S1. Summary of unit prices used to estimate household production and leisure time costs. **Table S2.** Summary of input parameters and uncertainty ranges for the economic models.

## Abbreviations

AUD: Australian dollars
ABS: Australian Bureau of Statistics
COPD: Chronic obstructive pulmonary disease
DALYs: Disability adjusted life years
FCA: Friction cost approach
GBD: Global Burden of Disease
HCA: Human capital approach
IVAWS: International Violence Against Women Survey
NHS: National health survey
IPV: Intimate partner violence
PAFs: Population attributable risk fractions
US: United States

## References

[1] Lim SS, Vos T, Flaxman AD, Danaei G, Shibuya K, Adair-Rohani H, AlMazroa MA (2012). A comparative risk assessment of burden of disease and injury attributable to 67 risk factors and risk factor clusters in 21 regions, 1990–2010: a systematic analysis for them Global Burden of Disease Study 2010. Lancet.

[2] Devries KM, Mak JYT, Garcia-Moreno C, Petzold M, Child JC, Falder G, Lim S, Bacchus LJ, Engell RE, Rosenfeld L (2013). Global health. The global prevalence of intimate partner violence against women. Science.

[3] Johnson H, Ollus N, Nevala S (2008). Violence against women: an international perspective.

[4] VicHealth (2004). The health costs of violence: measuring the burden of disease caused by intimate partner violence.

[5] Access Economics (2004). The cost of domestic violence to the Australian economy: Part 1.

[6] Garcia-Moreno C, Jansen H, Watts C, Ellsberg MC, Heise L (2005). WHO Multi-Country study on women’s health and domestic violence against women.

[7] Krug EG, Dahlberg TT, Mercy JA, Zwi AB, Lozano RE (2002). World report on violence and health.

[8] Schröttle M, Martinez M, Condon S, Jaspard M, Piispa M, Westerstrand J, Reingardiene J, Springer-Kremser M, Hagemann-White C, Brzank P (2006). Comparative reanalysis of prevalence of violence against women and health impact data in Europe – obstacles and possible solutions. Testing a comparative approach on selected studies. vol. 6th Framework Programme, Project No. 506348: Co-ordination Action on Human Rights Violations.

[9] Stene LE, Jacobsen GW, Dyb G, Tverdal A, Schei B (2013). Intimate partner violence and cardiovascular risk in women: a population-based cohort study. J Womens Health.

[10] Roman NV, Frantz JM (2013). The prevalence of intimate partner violence in the family: a systematic review of the implications for adolescents in Africa. Fam Pract.

[11] Australian Bureau of Statistics (2012). Personal safety survey, Cat. No. 4906.0.

[12] Jewkes R (2013). Intimate partner violence: the end of routine screening. Lancet.

[13] The National Council to Reduce Violence against Women and their Children. The cost of violence against women and their children. Canberra; 2009. p. 80.

[14] Cadilhac DA, Magnus A, Sheppard L, Cumming TB, Pearce DC, Carter R (2011). The societal benefits of reducing six behavioural risk factors: an economic modelling study from Australia. BMC Public Health.

[15] Black MC (2011). Intimate partner violence and adverse health consequences: implications for clinicians. Am J Lifestyle Med.

[16] Begg S, Vos T, Barker B, Stevenson C, Stanley L, Lopez AD (2007). The burden of disease and injury in Australia 2003.

[17] Rehm J, Taylor B, Patra J, Gerhard G (2006). Avoidable burden of disease: conceptual and methodological issues in substance abuse epidemiology. Int J Methods Psychiatr Res.

[18] Carbone-Lopez K, Kruttschnitt C, Macmillan R (2006). Patterns of intimate partner violence and their associations with physical health, psychological distress, and substance use. Public Health Rep.

[19] Australian Bureau of Statistics (2006). National Health Survey 2004–05, Cat. No. 4364.0.

[20] Anderson TM, Sunderland M, Andrews G, Titov N, Dear BF, Sachdev PS (2013). The 10-Item Kessler Psychological Distress Scale (K10) as a Screening Instrument in Older Individuals. Am J Geriatr Psychiatry.

[21] Australian Institute of Health and Welfare (2002). Health expenditure Australia 2000–01. Health and welfare expenditure series.

[22] Mathers C, Stevenson C, Carter R, Penm R. Disease costing methodology used in the Disease Costs abd Impact Study 1993–1994. AIHW; 1998. p. 96.

[23] Australian Bureau of Statistics (2008). How Australians use their time, 2006, Cat no. 4153.0.

[24] Australian Bureau of Statistics (2008). Average weekly earnings, May 2008 Cat no. 6302.0.

[25] Australian Bureau of Statistics (2008). Labour force Australia, Cat no. 6202.0.

[26] Cadilhac DA, Cumming TB, Sheppard L, Pearce DC, Carter R, Magnus A (2011). The economic benefits of reducing physical inactivity: an Australian example. Int J Behav Nutr Phys Act.

[27] Magnus A, Cadilhac D, Cumming T, Sheppard L, Pearce D, Carter R (2011). Economic benefits of achieving realistic smoking cessation targets in Australia. Am J Public Health.

[28] Magnus A, Cadilhac D, Cumming T, Sheppard L, Pearce D, Carter R (2011). The economic gains of achieving reduced alcohol consumption targets for Australia. Am J Public Health.

[29] Australian Institute of Health and Welfare (2008). National public health expenditure report 2005–06.

[30] Purchasing power parities. [http://www.oecd.org/std/prices-ppp/].

[31] Gold MR, Siegel JE, Russell LB, Weinstein MC (1996). Cost-effectiveness in health and medicine.

[32] Koopmanschap MA, Rutten FF (1996). A practical guide for calculating indirect costs of disease. Pharmacoeconomics.

[33] Liljas B (1998). How to calculate indirect costs in economic evaluations. Pharmacoeconomics.

[34] Australian Bureau of Statistics (2008). Time use survey: user guide 2006. Cat no. 4153.0.

[35] Australian Bureau of Statistics (2007). National survey of mental health and wellbeing: summary of results, Cat. No. 4326.0.

[36] World Health Organization (2013). Global and regional estimates of violence against women: prevalence and health effects of intimate partner violence and nonpartner sexual violence.

[37] Australian Bureau of Statisitcs (2013). Defining the data challenge for family, domestic and sexual violence: a conceptual data framework, report 4529.0.

[38] ABS Data Quality Framework, Cat. No. 1520.0. [http://www.abs.gov.au/ausstats/abs@.nsf/lookup/1520.0Main+Features1May+2009].

